# A Randomized, Double-Blind, Placebo-Controlled Clinical Trial on the Effect of a Dietary Supplement Containing Dry Artichoke and Bergamot Extracts on Metabolic and Vascular Risk Factors in Individuals with Suboptimal Cholesterol Levels

**DOI:** 10.3390/nu16111587

**Published:** 2024-05-23

**Authors:** Federica Fogacci, Marina Giovannini, Antonio Di Micoli, Giulia Fiorini, Elisa Grandi, Claudio Borghi, Arrigo F. G. Cicero

**Affiliations:** 1 Hypertension and Cardiovascular Risk Research Group, Medical and Surgical Sciences Department, Sant’Orsola-Malpighi University Hospital, 40138 Bologna, Italy; marina.giovannini3@unibo.it (M.G.); elisa.grandi@unibo.it (E.G.); claudio.borghi@unibo.it (C.B.); 2 Italian Nutraceutical Society (SINut), 40138 Bologna, Italy; 3 Cardiovascular Medicine Uniti, IRCCS Azienda Ospedaliero-Universitaria di Bologna, 40138 Bologna, Italy; antonio.dimicoli@aosp.bo.it (A.D.M.); giulia.fiorini@aosp.bo.it (G.F.)

**Keywords:** dietary supplement, nutraceutical compound, cholesterol, artichoke, bergamot

## Abstract

The aim of this study was to assess whether dietary supplementation with a nutraceutical blend comprising extracts of bergamot and artichoke—both standardized in their characteristic polyphenolic fractions—could positively affect serum lipid concentration and insulin sensitivity, high-sensitivity C-reactive protein (hs-CRP), and indexes of non-alcoholic fatty liver disease (NAFLD) in 90 healthy individuals with suboptimal cholesterol levels. Participants were randomly allocated to treatment with a pill of either active treatment or placebo. After 6 weeks, the active-treated group experienced significant improvements in levels of triglycerides (TG), apolipoprotein B-100 (Apo B-100), and apolipoprotein AI (Apo AI) versus baseline. Total cholesterol (TC), low-density lipoprotein cholesterol (LDL-C), non-high density lipoprotein cholesterol (Non-HDL-C), and hs-CRP also significantly decreased in the active-treated group compared to both baseline and placebo. At the 12-week follow-up, individuals allocated to the combined nutraceutical experienced a significant improvement in TC, LDL-C, Non-HDL-C, TG, Apo B-100, Apo AI, glucose, alanine transaminase (ALT), gamma-glutamyl transferase (gGT), hs-CRP, several indexes of NAFLD, and brachial pulse volume (PV) in comparison with baseline. Improvements in TC, LDL-C, Non-HDL-C, TG, fatty liver index (FLI), hs-CRP, and endothelial reactivity were also detected compared to placebo (*p* < 0.05 for all). Overall, these findings support the use of the tested dietary supplement containing dry extracts of bergamot and artichoke as a safe and effective approach for the prevention and management of a broad spectrum of cardiometabolic disorders.

## 1. Introduction

Regional and global prevalence of abnormal lipid levels (total cholesterol, TC > 190 mg/dL; 4.9 mmol/L) in the general population is high (39%) and increasing worldwide, reaching the highest values (54%) in Europe [1] A recently published report by the World Health Organization (WHO) has shed light on the urgent action needed to address this issue [1]. Estimates suggest that only one out of three hypercholesterolemic individuals have values of low-density lipoprotein cholesterol (LDL-C) within the recommended range [2]. Poor LDL-C control was estimated to be responsible of 3.9 million deaths worldwide (95% credible interval between 3.7 million and 4.2 million) [3]. Lifestyle improvement usually has a limited effect on cholesterolemia [4]. Statin treatment is indicated for the primary prevention of cardiovascular disease (CVD) in patients with moderate to high risk, but the risk–benefit ratio of lipid-lowering drugs in low-risk individuals is still debated [5].

Non-alcoholic fatty liver disease (NAFLD) has been widely associated with cardio-metabolic syndrome and its components [6]. It involves more than one third of the world’s adult population, and its management mainly consists of diet improvement and physically enhancing activity [7].

In this context, there is growing interest in identifying safe, new compounds potentially useful in improving both risk factors [8].

Polyphenols are naturally bioactive compounds present in plants and plant-derived products, as secondary metabolites [9].

The several biological effects of flavonoids derive from their chemical and biochemical properties, which have an impact on the composition of the microbiota and also regulate gene expression in chronic diseases while modulating various molecular pathways [10]. In fact, flavonoids have been shown to function as scavengers of free radicals, as well as exerting antioxidant, hepatoprotective, and anti-inflammatory effects [11]. Furthermore, combining data from various epidemiological studies and clinical trials, both total flavonoids and specific subclasses have been linked to a decreased occurrence of CVD, diabetes mellitus, and overall mortality [12]. In particular, a recent meta-analysis of 16 epidemiological studies including 462,194 adult participants with 23,473 death cases and a follow-up ranging from 4.8 to 28 years showed that flavonoid dietary intake was inversely associated with a total mortality relative risk reduction (RRR) of 13% (RR 0.87, 95% confidence interval (CI) 0.77–0.99) and a CVD mortality RRR of 15% (RR 0.85, 95%CI: 0.75–0.97) [13].

In recent times, standardized flavonoid extracts from artichoke and bergamot have been proposed as safe polyphenol-rich nutraceuticals for lowering lipid levels [14].

The phytocomplexes of bergamot and artichoke are very rich; in particular, referring to artichoke in addition to flavonoids, we have some relevant polyphenols such as caffeoylquinic acids (e.g., chlorogenic acid) that may contribute to the efficacy of the extracts [15]. Furthermore, we sought to assess whether dietary supplementation with a nutraceutical blend containing dried extracts of bergamot and artichoke could positively affect serum lipids concentration, C-reactive protein, some validated indexes of NAFLD, and endothelial reactivity in individuals with suboptimal cholesterol levels.

## 2. Materials and Methods

### 2.1. Trial Design and Participants

Our study had a randomized, double-blind, placebo-controlled, parallel-group design and enrolled Caucasian adult volunteers with suboptimal cholesterol levels recruited from the Outpatient Lipid Clinic of the “IRCCS Azienda Ospedaliero—Universitaria di Bologna” (Bologna University Hospital) in Italy.

Patients 18 to 70 years of age were eligible if they had LDL-C levels ranging from >115 mg/dL to <190 mg/dL, with triglyceride (TG) levels <400 mg/dL and an estimated 10-year cardiovascular risk of <5% according to the SCORE (Systematic Coronary Risk Evaluation) risk charts [16].

Exclusion criteria encompassed a history of CVD, obesity (body mass index (BMI) > 30 Kg/m^2^), diabetes mellitus, uncontrolled hypertension (systolic (SBP), and diastolic blood pressures (DBP) > 140/90 mmHg); positive test results for hepatitis B/C/E or human immunodeficiency virus; uncontrolled hypothyroidism; history of malignancies, liver disease, or renal disease; use of any medication or dietary supplement affecting blood pressure levels or serum lipids (particularly lipid-lowering drugs); alcoholism; pregnancy; and breastfeeding.

**Figure 1:**
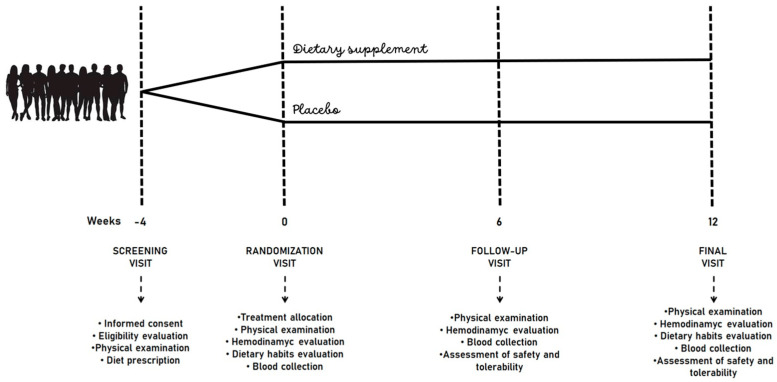
Study design.

Volunteers enrolled in the trial adhered to a low-fat, low-sodium Mediterranean diet for four weeks preceding randomization. Individuals were asked to increase vegetable intake, decrease salt in their diet, and reduce the consumption of processed meats, alcohol, and sweets. They were also encouraged to replace high-glycemic index carbohydrates with whole-grain foods, use extra-virgin olive oil as a substitute for butter, and low-fat dairy products and poultry as substitutes for high-fat dairy products and fat meats, respectively. The intervention period spanned 12 weeks. Following dispensation of the dietary supplement, patients attended clinic visits at 6 and 12 weeks. At each follow-up visit, patients underwent clinical assessment, including physical examination, laboratory analyses, and hemodynamic evaluations. The study timeline is shown in Figure 1.

The trial strictly adhered to the ethical principles outlined in the Declaration of Helsinki and The International Council for Harmonization of Technical Requirements for Registration of Pharmaceuticals for Human Use (ICH) Harmonized Tripartite Guideline for Good Clinical Practice (GCP). Its protocol received approval from the Ethical Committee of the University of Bologna (ClinicalTrial.gov ID: NCT06247137). In accordance with Good Clinical Practice guidelines, all patients provided written informed consent prior to their inclusion in the study.

### 2.2. Treatment

Following a one-month period of diet standardization, participants were randomly assigned to receive either an identical placebo pill or the active treatment, containing artichoke dry extract, bergamot dry extract, astaxanthin, chromium picolinate, and folic acid (Table 1).

Upon randomization, participants were furnished with boxes containing a total of nine blisters with ten tablets each. The tablets were manufactured and packaged by Meda Pharma S.p.A. (Monza, MB, Italy) in the facility in Confienza, Italy, following the European Good Manufacturing Practices (GMP), satisfying the requirements of the “Code Of Federal Regulation” title 21, volume 2, part 111, and the requirements of HACCP (Hazard Analysis and Critical Control Points).

Centralized randomization was conducted using computer-generated codes. Both participants and investigators were unaware of the group assignments. Randomization codes were securely stored in a sealed envelope, which remained unopened until after the completion of the study and statistical data analysis.

Throughout the study period, participants were directed to consume one pill of their designated treatment daily, ideally at a consistent time each day, preferably in the evening. At the conclusion of the trial, any remaining pills were collected for inventory purposes. Compliance among participants was evaluated by tallying the number of pills returned.

### 2.3. Assessments

#### 2.3.1. Clinical Data

Patients’ history encompassed inquiries regarding the presence of atherosclerotic CVD and other systemic conditions, allergies, and medication use. Validated, semi-quantitative questionnaires were used to collect information on individuals’ demographic characteristics, frequency and intensity of recreational physical activity, and dietary and smoking habits (i.e., distinguishing between former smokers, active smokers, or those who never smoked. If the respondent was a smoker, we recorded the number of years she/he had actively smoked and the number of cigarettes smoked on an average per day) [17].

Dietary data were assessed through an externally validated Food Frequency Questionnaire (FFQ) including several domains based on the daily and/or weekly consumption of a number of food groups, in accordance with the Mediterranean Diet [17]. The dietary composition and its variations throughout the study were analyzed using the MètaDieta^®^ software (INRAN/IEO 2008 revision/ADI), and all data were managed in accordance with company procedure IOA87.

Waist circumference (WC) was measured horizontally at the end of a normal expiration, at the midpoint between the lower margin of the last rib and the upper iliac crest. Height and weight were measured to the nearest 0.1 cm and 0.1 kg, respectively, with participants standing erect, eyes forward, wearing light clothing and bare feet. BMI was calculated by dividing body weight in kilograms by height squared in meters (Kg/m^2^).

#### 2.3.2. Laboratory Analyses

Biochemical analyses were conducted on venous blood samples collected after an overnight fast of at least 12 h with vials containing micronized silica and acrylic gel as clotting agents. Immediately following centrifugation, trained personnel performed laboratory analyses using standardized methods [17]. The following parameters were directly assessed: total cholesterol (TC), TG, high-density lipoprotein cholesterol (HDL-C), apolipoprotein B-100 (Apo B-100) and AI (Apo AI), glucose, creatinine, high-sensitivity C-reactive protein (hs-CRP), creatine phosphokinase (CPK), gamma-glutamyl transferase (gGT), alanine, and aspartate transaminase (ALT and AST).

LDL-C levels were determined using the Friedewald formula. Non-HDL-C was calculated by subtracting the HDL-C value from the TC value. The glomerular filtration rate (eGFR) was estimated using the chronic kidney disease epidemiology collaboration (CKD-epi) equation [18].

The lipid accumulation product (LAP) was computed as (WC − 65) × TG [mmol/L] for men and (WC − 58) × TG [mmol/L] for women [19]. The hepatic steatosis index (HSI) was derived from the 8 × AST/ALT ratio + BMI (+2 for women) [20]. The fatty liver index (FLI) was calculated as follows: [e^0.953 × ln (TG) + 0.139 × BMI + 0.718 × ln (GGT) + 0.053 × WC − 15.745^/(1  +  e^0.953 × ln (TG) + 0.139 × BMI + 0.718 × ln (GGT) + 0.053 × WC − 15.745^)] × 100 [21].

#### 2.3.3. Blood Pressure Measurements

The SBP and DBP were measured with a validated oscillometric device and a cuff of the appropriate size was applied on the right upper arm. To enhance the precision of detection, 3 BP readings were successively taken at intervals of 1 min from each other. The first reading was disregarded. The second and third readings were averaged and used as a study variable [22].

#### 2.3.4. Endothelial Reactivity

The endothelial function (EF) of arteries serves as a validated early indicator of atherosclerosis, representing the endothelial layer’s capacity to release nitric oxide (NO), which regulates smooth muscle tone within the conduit arteries’ arterial walls [23].

In accordance with the latest international guidelines [24], EF was assessed during the study using the Endocheck^®^ (BC Biomedical Laboratories Ltd., Vancouver, BC, Canada) that is included in the Vicorder^®^ device and generates highly reproducible measurements [25]. EF assessment was conducted with patients lying down, after a 12 h period of abstaining from smoking (including passive smoking) and caffeinated beverages. Following a 10 min rest period, brachial pulse volume (PV) waveforms were captured at baseline for 10 s and during reactive hyperemia. The BP cuff was then inflated to 200 mmHg for 5 min, after which PV waveforms were recorded for 3 min following cuff release. PV displacement was determined by calculating the percentage change in the PV waveform area, comparing waveforms before and during hyperemia through the equation PV2/PV1, where PV1 represents PV at the baseline and PV2 represents PV during hyperemia [26].

#### 2.3.5. Assessment of Safety and Tolerability

The safety and tolerability were continuously monitored throughout the study to identify any adverse events (AEs), including clinical safety assessments, laboratory results, vital signs, and physical examinations. A separate expert clinical event committee, appointed by the principal investigator, was blinded to classify AEs into categories of unrelated, unlikely related, possibly related, probably related, or definitely related to the treatment under investigation [27].

### 2.4. Statistical Analysis

Data were analyzed using intention-to-treat analysis with the Statistical Package for the Social Sciences (SPSS) version 21.0 for Windows.

The sample size was calculated for power of the study of 90% and an α error of 0.05. A total of 39 individuals per group were required to detect a mean change in LDL-C of 15 mg/dL at 12 weeks. To account for a dropout rate of 13%, we finally considered a sample size of 90 individuals (i.e., 45 individuals per arm).

The normal distribution of continuous variables was tested through the Kolmogorov–Smirnov test. Baseline characteristics of the study population were compared using the independent Student’s *t*-test and the χ^2^ test, followed by the Fisher’s exact test. Between-group differences were evaluated using repeated-measures analysis of variance (ANOVA), followed by Tukey’s post hoc test. Results were presented as means and standard deviations. All statistical tests were two-sided, and a significance level of *p*-value < 0.05 was considered as statistically significant.

## 3. Results

### 3.1. Efficacy Analysis

**Figure 2:**
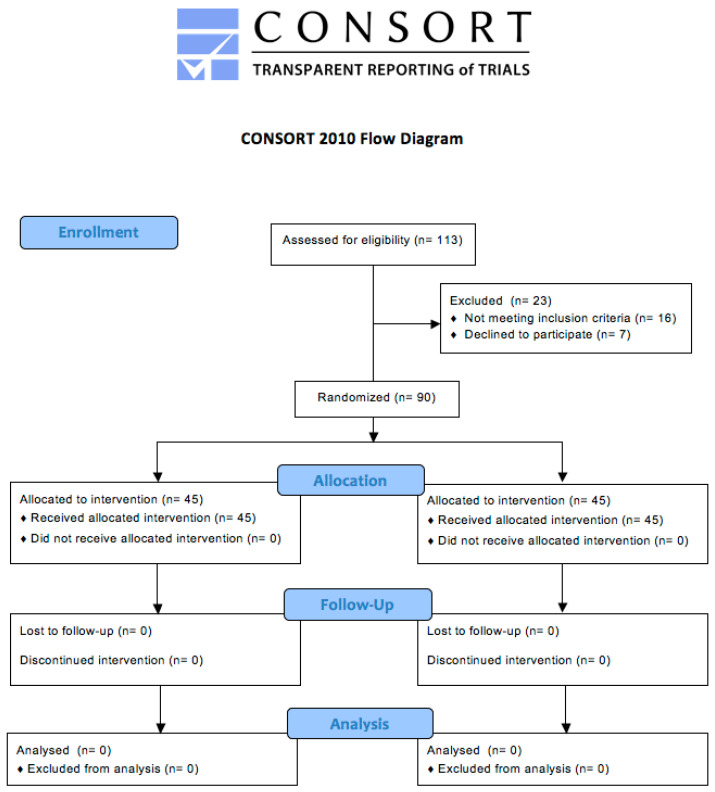
Consolidated Standards of Reporting Trails (CONSORT) flow diagram of the progress through the phases of the clinical study. The groups were balanced, and no individuals were lost to follow-up in either study arm.

A total of 113 individuals were screened, and 90 volunteers underwent randomization from January to November 2020. All subjects who were enrolled in the trial successfully completed the study according to its protocol (Figure 2).

Overall compliance (as assessed at the end of the treatment period) was 98% in the active treatment group and 96% in the placebo group. The final sex-distribution did not reveal any significant between-group differences (N = 23 in the active treatment group, N = 24 in the placebo group; χ^2^ = 1.542, *p*-value > 0.05). No statistically significant alterations were observed in participants’ dietary habits from randomization to the end of the study, including changes in total energy and macronutrient intake (Table 2).

At the outset, study groups were adequately balanced on the measured covariates (Table 3). The efficacy analysis at week 6 provided evidence that dietary supplementation with artichoke and bergamot dried extracts significantly decreases TG, Apo B-100, and Apo AI compared to baseline and TC, LDL-C, Non-HDL-C, and hs-CRP compared to baseline and placebo (Table 3). The significant effects were sustained and improved over time. However, no significant change was observed between the 6-week and 12-week follow-up in any of the investigated parameters.

At the end of the trial, dietary supplementation with the tested, combined nutraceutical was associated with significant improvements in TC, LDL-C, Non-HDL-C, TG, Apo B-100, Apo AI, fasting glucose, ALT, gGT, LAP, HIS, FLI, hs-CRP, and PV, compared to baseline, and with significant reductions in TC, LDL-C, Non-HDL-C, TG, FLI, and hs-CRP compared to placebo (Table 3). DBP decreased only slightly in the placebo group, without reaching statistical significance (*p*-value > 0.05).

### 3.2. Safety Analysis

The clinical study was successfully completed by all enrolled volunteers in accordance with the study’s design, resulting in a dropout rate of 0%. Treatment-emergent AEs were reported in 2% of patients overall and were always transient and mild in severity. One patient allocated to the active treatment experienced dyspepsia; one patient in the placebo group experienced diarrhea. No laboratory abnormalities occurred during the trial.

## 4. Discussion

Over the past few decades, there has been an increasing interest in exploring the efficacy of natural compounds that target various biochemical pathways simultaneously. [28]. Within this framework, dietary polyphenols have garnered notable interest [29].

A growing body of evidence suggests that standardized artichoke and bergamot flavonoid extracts affect lipid profiles in individuals with suboptimal cholesterolemia, leading to their promotion as safe lipid-lowering agents [30,31,32].

Their lipid-lowering mechanism of action seems to be at least additive, if not synergistic, so that their combined use could be of particular interest [33].

In animal studies, artichoke flavonoids have been shown to impede the biosynthesis of cholesterol from 14-C-acetate, likely by inhibiting the enzyme 3-hydroxy-3-methylglutaryl-coenzyme A (HMG-CoA) reductase [34]. Additionally, these flavonoids interact with liver sterol regulatory element-binding proteins (SREPBs) and acetyl-CoA C-acetyltransferase (ACAT), leading to an increase in the fecal excretion of bile acids [35]. Bergamot flavonoid extract also exerts a strong dose-dependent inhibition of HMG-CoA reductase [36]. However, many different lipid-lowering mechanisms have been attributed to bergamot flavonoids, from cholesterol absorption inhibition in the bowel to inhibition of the AMP-kinase [37].

From a clinical point of view, the lipid lowering effect of both artichoke and bergamot extracts have been clearly defined. Among polyphenols, the active components of bergamot belong to the family of flavonoids, but instead in artichoke, we have a combination of flavonoids, caffeoylquinic acids, and chlorogenic acid [15]. In particular, a meta-analysis of nine randomized clinical trials on the effects of artichoke extract on plasma lipids showed TC (weighted mean difference (WMD): −17.6 mg/dL, 95%CI: −22.0 to −13.3, *p* < 0.001), LDL-C (WMD: −14.9 mg/dL, 95%CI: −20.4 to −9.5, *p* = 0.011) and TG (WMD: −9.2 mg/dL, 95%CI: −16.2 to −2.1, *p* < 0.011), without a significant change in HDL-C concentrations [38]. Moreover, a further recent meta-analysis of 14 randomized clinical trials has concluded that bergamot extracts are able to decrease TC (WMD: −63.6 mg/dL; 95%CI: −78.0 to −49.2; *p* < 0.001), TG (WMD: −74.7 mg/dL; 95%CI: −83.6 to −65.9; *p* < 0.001), LDL-C (WMD: −55.4 mg/dL; 95%CI: −67.3 to −43.6; *p* < 0.001) and increase HDL-C (WMD: 5.8 mg/dL; 95%CI: 3.3 to 8.3; *p* < 0.001), respectively, in humans [39]. However, in both cases, the best lipid-lowering effect has been obtained with more than one tablet per day [40]. On the other hand, currently, there is no specific evidence related to the simultaneous supplementation with these nutraceutical compounds.

In the present study, dietary supplementation with a combination of standardized artichoke and bergamot dry extracts was effective in lowering a number of lipoprotein fractions and apolipoprotein B, with additional significant effects on hs-CRP and some NAFLD indexes. These data are in line with those obtained with other dietary supplements containing polyphenolic fractions of both artichoke dry extract and bergamot but require the administrations of two pills per day [41,42]. At the end of the trial, the dietary supplementation also resulted in a significant improvement in PVC as a marker of endothelial reactivity. A similar result was observed in a small clinical trial where endothelial function was measured in terms of reactive hyperemia [43].

According to findings from the Cholesterol Treatment Trialists’ (CTT) meta-analyses of statin trials, there exists a direct correlation between lowering LDL-C levels and reduced occurrences of atherosclerotic CVD events. In particular, a 1 mmol/L (~39 mg/dL) LDL-C reduction has been associated with a 21–23% relative risk reduction in CVD events over 5 years [44]. Strong and increasing evidence indicates that this linear relationship is observed regardless of the approach used to lower LDL-C. (i.e., low-fat diet, anion exchange resins, statins, ezetimibe, and/or new generation lipid-lowering drugs) [45]. Moreover, a meta-analysis of 35 trials with 17280 participants concluded that a 1% increase in endothelial reactivity is associated with a 12% RRR of CVD events (adjusted RR 0.88 [0.84–0.91], *p* < 0.001) [46]. Furthermore, considering the findings of the present study, it is plausible to expect a 9–11% CVD event reduction after long-term dietary supplementation with the tested combined nutraceutical.

In this trial, hsCRP, a known marker and predictor of CV events [47], decreased by 11%, a similar effect to that observed following dietary supplementation with polyunsaturated fatty acids (PUFAs) [48]. Fasting glucose only improved in the active treatment group versus placebo. This observation could be at least partly due to a small amount of chromium contained within the tested dietary supplement. In effect, the glucose-lowering effect of chromium has already been clearly demonstrated in several randomized clinical trials [49].

HDL-C and Apo AI improved only in the actively treated group, but the changes did not reach statistical significance compared to the placebo. Further studies should be carried out to confirm if these observations could further affect the potential preventive effect of the tested nutraceutical.

Despite the positive findings and the possible practical implications, this study has some limitations. We acknowledge the relatively small sample size, even though the study was adequately powered for the primary outcomes. Of course, evaluation of the impact of the tested dietary supplement across age or sex would have potentially been of interest, but the study was not designed for this aim and is currently underpowered for this kind of subanalysis. Moreover, the relatively short follow-up does not clarify the possible occurrence of adaptation phenomena, which, however, have never been documented for polyphenols before. In fact, in this trial, the results achieved after six weeks of treatment were confirmed or only mildly improved (but not impaired) after further six weeks of follow-up. At the same time, the short duration of the study does not allow us to measure effects on hard outcomes. However, a strong and growing body of literature supports the concept that persistent lower LDL-C in blood as well as a persistent reduction in plasma LDL achieved with different pharmacological tools are always associated with a proportional decreased risk of cardiovascular (mainly coronary) disease. In particular, a milestone Mendelian randomization study by Ference BA et al. showed that long-term exposure to (even mildly) lower LDL-C mediated by nine polymorphisms in six different genes is associated with a 54.5% (95% confidence interval: 48.8% to 59.5%) reduction in the risk of coronary artery disease for each 38.7 mg/dL) of lower LDL-C [50] On the other side, another large meta-analysis carried out on data from 312,175 patients with a mean baseline LDL-C of 122.3 mg/dL from 49 trials with 39 645 major vascular events showed a risk reduction for major vascular events per 38.7 mg/dL reduction in LDL-C level of 0.77 (95%CI, 0.71–0.84; *p* < 0.001) for statins and 0.75 (95%CI, 0.66–0.86; *p* = 0.002) for established non-statin interventions that work primarily via the upregulation of LDL receptor expression (among which diet) (between-group difference, *p* = 0.72) [45]. It could be argued that, in our study, the placebo performed well versus baseline. However, following dietary supplementation with dried artichoke and bergamot extracts, the investigated cardiometabolic parameters improved more than following the placebo. Moreover, this is a confirmation of the effectiveness of the dietary education that was carried out in both experimental groups during the study. Another limitation of the study is that liver ultrasound to confirm the diagnosis of NAFLD was not performed. Anyway, data analysis on NAFLD biomarkers was a secondary explorative objective, since NAFLD was not an inclusion criterion of the trial. It is crucially important that longer-term clinical trials clarify whether the observed treatment-dependent changes in liver function tests will correspond to a meaningful improvement in hepatic stiffness/fibrosis. Finally, we cannot exclude the possible contribution of non-phenolic compositions of bergamot and artichoke extracts on their final effect on the investigated parameters. This is a main methodological issue seen in many clinical studies testing the effect of plant or food based dietary supplements. Nevertheless, the test was carried out against a placebo, and the observed results are in line with the previous literature. On the other hand, minor components of the combined dietary supplement (astaxanthin, folate, chromium picolinate) could also have exerted some biological effects. However, they would be hardly responsible for the observed changes in lipid parameters (because of lack of effects of these compounds on these parameters) and endothelial function (mainly because of their low dosage).

Further research is necessary to delve into the underlying reasons and mechanisms behind the positive effects recorded during the trial. For example, the impact on serum lipids and inflammation could be partly influenced by alterations to the gut microbiota induced by polyphenol supplementation. In fact, some experimental data suggest that supplementation with bergamot and artichoke polyphenols may exert a beneficial effect on the composition of the gut microbiota [10,51].

## 5. Conclusions

The study shows that dietary supplementation with polyphenolic fractions of both dry artichoke and bergamot extracts safely exerts significant improvements in serum lipids, systemic inflammation, indexes of NAFLD, and endothelial reactivity in healthy individuals with suboptimal cholesterolemia. These findings collectively support the use of a dietary supplement made of standardized, dry artichoke and bergamot extracts in clinical practice.

## Figures and Tables

**Table 1:** Quantitative composition of the food supplement used in the clinical study.

Ingredients	Quantity per Tablet
Artichoke dry extract (*Cynara scolymus* L.)	700 mg
equivalent to caffeoylquinic acids	52.5 mg
chlorogenic acid	35 mg
flavonoids	11.9 mg
Bergamot dry extract (Citrus bergamia Risso & Poit)	375 mg
equivalent to flavonoids	150 mg
Astaxanthin-rich oleoresin from Haematococcus pluvialis algae	20 mg
equivalent to astaxanthin	0.5 mg
Chromium picolinate	320 µg
equivalent to chromium	40 µg
Folic acid	200 µg

**Table 2:** Diet composition at enrollment and at the end of the clinical trial. Values are reported as mean ± standard deviation.

Parameters	Dietary Supplement Containing Artichoke and Bergamot Dry Extracts (*N.* 45)		Placebo (*N.* 45)	
	Baseline	12-Week Follow-Up	Baseline	12-Week Follow-Up
Total energy (Kcal/day)	1499 ± 119	1501 ± 125	1494 ± 111	1486 ± 129
Carbohydrates (% of total energy)	54.2 ± 2.7	52.2 ± 2.6	53.5 ± 3.1	53.4 ± 2.8
Proteins (% of total energy)	18.1 ± 1.5	17.4 ± 1.9	18.3 ± 1.9	18.5 ± 1.7
Animal protein (% of total energy)	10.5 ± 0.9	9.7 ± 0.8	10.6 ± 1.1	10.9 ± 0.7
Vegetal protein (% of total energy)	6.9 ± 0.7	7.3 ± 0.9	6.6 ± 0.6	6.8 ± 0.8
Total fats (% of total energy)	28.7 ± 2.4	28.5 ± 2.5	28.2 ± 1.6	29.4 ± 2.2
Saturated fatty acids (% of total energy)	8.9 ± 0.8	9.2 ± 0.7	9.3 ± 0.7	8.8 ± 0.9
MUFA (% of total energy)	12.6 ± 0.9	12.2 ± 1.0	12.8 ± 1.1	12.3 ± 1.2
PUFA (% of total energy)	6.7 ± 0.6	6.3 ± 0.4	6.6 ± 0.4	6.8 ± 0.5
Total dietary fibers (g/day)	17.9 ± 1.5	18.1 ± 1.8	18.1 ± 1.5	17.7 ± 1.4
Cholesterol (mg/day)	193.2 ± 14.3	188.2 ± 13.3	193.9 ± 13.5	195.8 ± 13.7

MUFA = monounsaturated fatty acids; N = number of individuals; PUFA = polyunsaturated fatty acids.

**Table 3:** Anthropometric, hemodynamic, and blood chemistry parameters from the baseline to the end of the clinical trial, expressed as mean ± standard deviation.

Parameters	Dietary Supplement Containing Artichoke and Bergamot Dry Extracts (*N.* 45)			Placebo (*N.* 45)		
	Baseline	6-Week Follow-Up	12-Week Follow-Up	Baseline	6-Week Follow-Up	12-Week Follow-Up
Age (years)	46.2 ± 3.7	-	-	47.3 ± 3.2	-	-
BMI (Kg/m^2^)	26.9 ± 1.9	26.6 ± 1.5	26.4 ± 1.4	26.8 ± 1.7	26.8 ± 1.6	26.6 ± 1.7
SBP (mmHg)	126 ± 9	126 ± 7	125 ± 6	128 ± 8	127 ± 9	129 ± 9
DBP (mmHg)	84 ± 7	85 ± 5	84 ± 4	85 ± 6	83 ± 6	83 ± 5
Total Cholesterol (mg/dL)	245 ± 13	207 ± 10 *°	205 ± 9 *°	238 ± 12	229 ± 10	228 ± 12
LDL-C (mg/dL)	157 ± 12	136 ± 7 *°	132 ± 6 *°	161 ± 9	154 ± 10	154 ± 8
HDL-C (mg/dL)	44 ± 3	45 ± 4	47 ± 3 *	45 ± 2	45 ± 3	45 ± 3
Non-HDL-C (mg/dL)	211 ± 13	162 ± 9 *°	158 ± 8 *°	203 ± 11	184 ± 11 *	183 ± 10 *
Triglycerides (mg/dL)	151 ± 13	132 ± 9 *	129 ± 7 *°	159 ± 12	142 ± 11 *	140 ± 9 *
Apolipoprotein B100 (mg/dL)	127 ± 9	115 ± 5 *	117 ± 7 *	124 ± 8	121 ± 7	123 ± 8
Apolipoprotein AI (mg/dL)	142 ± 12	148 ± 8 *	149 ± 9 *	145 ± 11	147 ± 10	146 ± 9
Fasting glucose (mg/dL)	87 ± 6	84 ± 6	82 ± 5 *	90 ± 9	88 ± 9	87 ± 10
AST (U/L)	18 ± 6	17 ± 3	16 ± 3	21 ± 6	22 ± 5	21 ± 4
ALT (U/L)	21 ± 5	19 ± 5	17 ± 6 *	23 ± 7	23 ± 4	22 ± 5
gGT (U/L)	25 ± 7	22 ± 6	21 ± 4 *	28 ± 9	26 ± 6	25 ± 6
Lipid Accumulation Product	63 ± 12	60 ± 9	58 ± 9 *	64 ± 14	63 ± 11	63 ± 9
Hepatic Steatosis Index	37 ± 5	36 ± 4	35 ± 4 *	38 ± 5	37 ± 4	37 ± 3
Fatty Liver Index	55 ± 9	52 ± 9	51 ± 7 *°	57 ± 11	56 ± 10	56 ± 9
CPK (U/L)	129 ± 37	118 ± 27	122 ± 22	134 ± 42	142 ± 28	139 ± 31
eGFR (mL/min)	83.9 ± 3.3	84.2 ± 3.7	84.5 ± 2.8	84.3 ± 3.4	85.3 ± 3.6	83.9 ± 3.9
hs-CRP (mg/L)	3.01 ± 0.22	2.71 ± 0.16 *°	2.68 ± 0.17 *°	2.90 ± 0.25	2.82 ± 0.32	2.87 ± 0.29
Pulse Volume (%)	63.9 ± 6.3	64.8 ± 4.8	69.2 ± 3.7 *°	65.4 ± 6.1	66.5 ± 5.1	64.1 ± 4.5

* *p*-value < 0.05 versus baseline; ° *p*-value < 0.05 versus placebo. ALT = alanine aminotransferase; AST = aspartate aminotransferase; BMI = body mass index; CPK = creatine phosphokinase; DBP = diastolic blood pressure; eGFR = estimated glomerular filtration rate; gGT = gamma-glutamyl transferase; HDL-C = high-density lipoprotein cholesterol; hs-CRP = high sensitivity C-reactive protein; LDL-C = low-density lipoprotein cholesterol; N = number of individuals; SBP = systolic blood pressure.

## Data Availability

Data supporting the study’s findings are available from the corresponding author with the permission of the University of Bologna.
